# Successful Use of Myosure in the Management of Cesarean Scar Ectopic Pregnancy

**DOI:** 10.7759/cureus.17500

**Published:** 2021-08-27

**Authors:** Naveena R Daram, Lawrence Berry, Mona Fakih, Ali Alhousseini

**Affiliations:** 1 Obstetrics and Gynecology, Oakland University William Beaumont School of Medicine, Rochester, USA; 2 Radiology, Wayne State University School of Medicine, Detroit, USA; 3 Obstetrics and Gynecology, Beaumont Health, Royal Oak, USA; 4 Maternal and Fetal Medicine, Beaumont Health, Royal Oak, USA

**Keywords:** cesarean scar, ectopic pregnancy, myosure, hysteroscopy, cesarean scar pregnancy

## Abstract

Cesarean scar pregnancy (CSP) is a very serious complication of a prior cesarean delivery. The major risks associated with CSP are uncontrolled hemorrhage and uterine rupture, potentially leading to future infertility. Management of CSP remains a major obstetric challenge without a well-defined therapeutic procedure. Dilation & curettage is a commonly used procedure for the treatment of CSP. However, it can be ineffective and often leads to definite infertility. Therefore, we present a case of the successful use of an alternative procedure, Myosure® hysteroscopy, in the treatment of CSP. We herein report the case of a 32-year-old G5P3013 woman who presented with vaginal bleeding and past history of three cesarean sections. She was found to have a CSP with fetal pole and cardiac activity at 6 weeks 2 days. The patient was initially treated with a systemic methotrexate injection, but there was persistence of cardiac activity. A second course of methotrexate was administered into the gestational sac, which systemically led to successful fetal cardiac arrest and downtrend of beta-human chorionic gonadotropin (HCG) level. A dilation & curettage procedure was not successful in removing products of conception. A Myosure hysteroscopy procedure, however, was successful in removing products of conception. The patient was discharged after a negative ultrasound and beta-HCG level. In our review of the literature, we found that there is no general consensus on the management of cesarean scar ectopic pregnancies. To date, there is no literature cited about the use of Myosure for cesarean scar ectopic pregnancies. However, our case suggests that Myosure can be effective for CSP and this warrants a larger-scale controlled study to better evaluate this as a treatment for this condition.

## Introduction

Cesarean scar ectopic pregnancy was first published in the literature in 1978 [1]. It has since been discovered that the incidence of cesarean scar ectopic pregnancy ranges from approximately 1/800 to 1/2500 of all pregnancies [2,3]. With the rise in cesarean deliveries, the incidence of cesarean scar pregnancy is increasing in parallel [4]. Though this is a rare complication of pregnancy, it is one that can be potentially fatal to the mother without adequate management, leading to hemorrhage due to uterine rupture and subsequent death [5].

Slight vaginal bleeding is the most common symptom of women presenting with a cesarean scar pregnancy, with abdominal discomfort the second leading symptom that patients experience [6]. However, many women may also present asymptomatic at the time of diagnosis [5]. Transvaginal ultrasound remains the main imaging study to investigate and diagnose a cesarean scar ectopic pregnancy. A transabdominal ultrasound may also be used in conjunction for more panoramic views [7].

Treatment depends largely on the presentation of the patient. Though there is no consensus on a preferred mode of treatment for patients with cesarean scar ectopic pregnancy, the aim of removing the gestational sac and ectopic pregnancy in order to preserve the fertility of the woman remains [8]. Management of the patient may include medical treatment with methotrexate or through surgical techniques. Cervical dilation & curettage (D&C) is one of the most commonly used methods for cesarean scar ectopic pregnancy, mainly due to its low costs [8]. However, it can often fail to remove all of the fetal tissue from the uterus and cause further complications such as decreased fertility.

This case report describes a woman with cesarean scar ectopic pregnancy who had multiple treatments for removal of the gestational sac. She underwent methotrexate injection twice with failure both times. This was followed by a D&C that also led to incomplete removal of the fetal tissue. She finally underwent Myosure hysteroscopy to remove the fetal products with great success. A hysteroscopy is a procedure in which you have direct visualization of the uterine cavity with a Myosure device able to perform the tissue collection. This procedure provides a technical advantage to D&C in that Myosure hysteroscopy is done under direct visualization but a D&C is a blind procedure. Myosure is classically used for the removal of uterine fibroids and polyps, but has not yet been cited for use in cesarean scar ectopic pregnancies. This case provides an example of how this technique can be used successfully for the management of cesarean scar ectopic pregnancies.

## Case presentation

The patient is a 32-year-old G5P3013 who presented with vaginal bleeding and abdominal pain radiating to the back at 6 weeks 0 days gestational age (calculated from last menstrual period). She has history of one vaginal delivery with fetal demise and three prior cesarean sections abroad in Iraq, all of which were a low transverse incision. The patient states that the last cesarean section was a difficult procedure due to the thin nature of her uterine wall. The patient has no prior history of ectopic pregnancy. There is no other significant past or family medical history.

Hospital course

**Figure 1:**
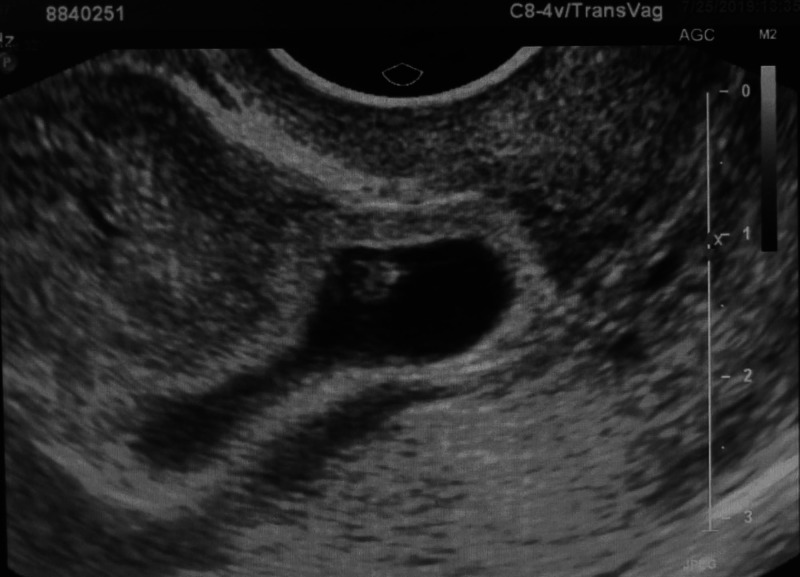
Transvaginal ultrasound showing cesarean scar ectopic pregnancy at 6 weeks and 3 days.

During the patient’s initial presentation, lab studies revealed her beta-human chorionic gonadotropin (HCG) levels to be 49,429 mIU/mL. The patient underwent a transvaginal and transabdominal ultrasound, which showed a cesarean scar pregnancy with a present cardiac activity of 101 BPM in a single gestational sac located in the mid-uterus (Figure 1). Crown rump length was found to be 3.8 mm. At this time, the patient underwent treatment with methotrexate. However, beta-HCG levels were trending upwards during her follow-up appointment the next morning (62,486 mIU/mL) with persistent symptoms of vaginal bleeding and abdominal pain. Moreover, fetal heart activity was still detected on transvaginal ultrasound. A repeat course of methotrexate injection, both intramuscular and into the gestational sac, was performed. However, transvaginal ultrasound two days later still revealed present cardiac activity in the fetal pole. At this time, the plan was to observe the patient’s follow-up ultrasounds and beta-HCG levels in the next few days. Subsequent ultrasounds revealed now absent cardiac activity (fetal demise) but retained fetal products of conception in the cesarean scar. The patient underwent a D&C procedure after being informed about the potential for infertility following it. Several days later, the patient once again presented with vaginal bleeding after the procedure. Ultrasound revealed a complex fluid collection in the endometrium, with a thickened endometrial layer, most likely due to retained products of conception. She then underwent the final management strategy for this condition, a Myosure hysteroscopy procedure. A 0-degree custom hysteroscope was introduced under direct visualization and the Myosure was placed through the operative port. Products of conception were suctioned through the port until the uterine cavity was clear of all ectopic tissue. No perforation was noted and there were no subsequent complications from this procedure.

Outcome and follow-up

Ultrasound following the Myosure hysteroscopy procedure revealed to be normal with no remaining products of conception. Negative beta-HCG levels of the patient confirmed this. The patient was discharged and given a follow-up gynecology appointment.

## Discussion

Ectopic pregnancies are a potentially life-threatening complication that can occur in up to 1-2% of all pregnancies [9]. The most common location of an ectopic pregnancy is in the ampulla of or isthmus of the fallopian tube [10]. Rarely, an ectopic pregnancy can occur inside of a cesarean scar if the woman has a past history of cesarean section(s). This can be fatal to the mother if the cesarean pregnancy were to rupture and hemorrhage. If left untreated, this could also lead to infertility in the patient. Therefore, prompt diagnosis and management of a cesarean scar ectopic pregnancy is absolutely crucial, especially since the patient may present asymptomatic in many cases.

The management of this condition is dependent on the presentation of the patient. Often times, there is no consensus on the best route of treatment. D&C is a common procedure undertaken for the management of cesarean scar ectopic pregnancy. However, as we have described in this case, it can often be ineffective. Therefore, our case presents a different management method for this type of ectopic pregnancy - the use of Myosure hysteroscopy. The use of hysteroscopy for the treatment of this cesarean scar pregnancy has been cited a few times in the literature thus far [11,12]. The use of Myosure has been cited once for the management of cornual ectopic pregnancy [10]. However, to our knowledge, this is the first case in which a Myosure device was used as a successful treatment for cesarean scar ectopic pregnancy. 

## Conclusions

Women with a history of cesarean deliveries are at an increased risk of developing cesarean scar ectopic pregnancies. These pregnancies can be a potentially fatal complication for the mother. Unfortunately, there is no general consensus about the preferred mode of treatment for this condition. Myosure hysteroscopy procedure is a minimally invasive procedure that can be used for the removal of gestational products in a cesarean scar ectopic pregnancy with good success. From the success of our case, we are suggesting that we move toward making this technique a first-line treatment for cesarean scar ectopic pregnancies, mainly due to the advantage of direct visualization as compared to D&C. Myosure's safety profile has also been ensured by its common usage in other conditions thus far, such as for the removal of uterine polyps and fibroids. We now recommend a more controlled study in order to evaluate the true efficacy of using Myosure as a treatment for this condition. 
